# Multi-Response Parameter Interval Sensitivity and Optimization for the Composite Tape Winding Process

**DOI:** 10.3390/ma11020220

**Published:** 2018-01-31

**Authors:** Bo Deng, Yaoyao Shi, Tao Yu, Chao Kang, Pan Zhao

**Affiliations:** The Key Laboratory of Contemporary Design and Integrated Manufacturing Technology, Ministry of Education, Northwestern Polytechnical University, Xi’an 710072, China; dengbo1025@outlook.com (B.D.); scor00@163.com (T.Y.); kaochao_017@163.com (C.K.); pan.zhao@hotmail.com (P.Z.)

**Keywords:** composite tape winding process, tensile strength, void content, sensitivity analysis, interval optimization

## Abstract

The composite tape winding process, which utilizes a tape winding machine and prepreg tapes, provides a promising way to improve the quality of composite products. Nevertheless, the process parameters of composite tape winding have crucial effects on the tensile strength and void content, which are closely related to the performances of the winding products. In this article, two different object values of winding products, including mechanical performance (tensile strength) and a physical property (void content), were respectively calculated. Thereafter, the paper presents an integrated methodology by combining multi-parameter relative sensitivity analysis and single-parameter sensitivity analysis to obtain the optimal intervals of the composite tape winding process. First, the global multi-parameter sensitivity analysis method was applied to investigate the sensitivity of each parameter in the tape winding processing. Then, the local single-parameter sensitivity analysis method was employed to calculate the sensitivity of a single parameter within the corresponding range. Finally, the stability and instability ranges of each parameter were distinguished. Meanwhile, the authors optimized the process parameter ranges and provided comprehensive optimized intervals of the winding parameters. The verification test validated that the optimized intervals of the process parameters were reliable and stable for winding products manufacturing.

## 1. Introduction

Composite prepreg tape winding technology is an effective way to fabricate rotationally composite materials, especially in the field of aerospace motor manufacture. Due to the superior strength to density ratio, the prepreg tape winding process has been widely used to fabricate solid rocket motor nozzle, ablation resistance and heat protection material parts, launch tube, and special equipment for aerospace aircraft [1,2,3,4]. The prepreg tape, a woven fabric consisting of fiber and resin, can be classified by thermoset and thermoplastic matrix systems. Today’s applications are mainly manufactured with thermoset matrix systems [5]. Although thermoplastic matrix materials have some advantages like good impact resistance, lower process costs, and recyclable character, thermoset matrix materials are still extensively employed to fabricate aviation products for their predominant ablation resistance and corrosion resistance. According to the literature [5,6,7,8,9,10,11], processing parameters, such as heating temperature, tape tension, roller force, and winding speed, play a crucial role in the composite tape winding process. As typical representative performances of composite products, void content and tensile strength can be used to measure the physical and mechanical properties of tape winding products, respectively. Meanwhile, a more reasonable parameters combination and optimized variables interval will not only help to improve the physical performance of products, but also be beneficial to improve the mechanical properties.

The last few decades have witnessed a rapid development in composite prepreg tape winding technology. The tape winding process shows great potential for efficient manufacturing of high strength composite structures. W. Polini et al. [12] discussed the influence of the main winding parameters on tension with robotized filament winding technology. Their study determined both the geometric parameters characterizing the winding trajectory and the winding speed that allows the winding tension to be kept near to the nominal value of good composite parts. F. Chinesta et al. [13] calculated the temperature and the mechanical field of the tape placement process and then evaluated the residual stresses of tape placement products. They also analyzed the induced distortion by solving the associated elastic problem at the structural level with the obtained residual stresses. In Mikhael Tannous’s paper [2], a finite element model was proposed for the simulation of the thermo-mechanical tape winding process, and the results showed that mechanical factors and friction play an important role in the winding process. Qing Yu Cui et al. [14] analyzed the function of tension by measuring the physical and mechanical properties of specimens, and considered that the wrinkles and waves on the surface of the T300/epoxy bearing composites can be eliminated using the proper method. The importance of tape placement process parameters was discussed, initially, in Jinxiang Cheng’s article [15]. Subsequently, a genetic algorithm combined with multi-objective optimization theory was applied to determine an optimum set of factors for obtaining the desired composite components with high speed and best layup quality. Muhammad Amir Khan et al. [16] revealed the relationship between tape placement process parameters with the interlaminar bond degree with a simulation method. Their paper showed that extended consolidation efforts were required to flatten the rough tape in order to facilitate the polymer diffusion. Similarly, W.J.B. Grouve et al. [17] investigated experimentally the effect of the tape placement processing parameters on the bond strength using a mandrel peel test. Muhammad Amir Khan et al. [18] studied the process sensitivity on individual parameters of the thermoplastic tape placement and identified the main parameters governing the resulting laminate quality. They thought that large roller diameters or consolidation lengths could only benefit in combination with higher forces. Nayani Kishore Nath [19] demonstrated that optimization of the process parameters of the tape winding process would achieve better insulative resistance using Taguchi’s robust design methodology and provided the optimum set of control factors for the manufacturing of nozzle throat back up liners. In the paper of Khan et al. [20], simulated void distribution through thickness and density were compared with measured values to trace the affecting input parameters. They found additional repetitive passes with hot gas and consolidation roller force contributed to the reduction of void content in most top layers. In S. Paciornik’s work [21], digital microscopy was used to characterize the microstructure of fiber-reinforced composite tubes manufactured by filament winding. B.N. Fedulov et al. [22] researched the effects of manufacturing defects of laminate composite materials on the first ply failure load and ultimate strength of the laminate and gave a conclusion that tape waviness results in an almost linear relationship between strength reduction and the waviness height ratio in cases of compression loading but did not affect the laminate strength under tension loading. Kaven Croft [23] and his collaborators investigated the effect of four principal defect types on ultimate strengths and provided the conclusion that ultimate strength was less affected by the different defect configurations at the lamina level (overall less than 5%) as opposed to the laminate level (up to 13%). Roham Rafiee [24] developed a suitable modeling procedure to predict the longitudinal and hoop tensile strength of glass reinforced polyester pipes incorporating sand as a core material. Tsugiyuki Okuya et al. [4] produced a co-cured carbon fiber reinforced polymer (CFRP) strand filament-wound specimen with end tabs to improve the tensile strength and bonding strength of the end grips with CFRP strand.

To sum up, a large number of literature papers have focused on process modeling, performance control, and the parameters for optimizing the composite tape winding process. Nevertheless, these works are short of sensitive analysis about the tape winding process parameters and in-depth study concerning interval optimization at the multi objects level. In fact, due to many uncontrollable factors occurring in the actual manufacturing procedure, a relative effective process parameter interval may be more popular with operators than the detailed parameter number. Moreover, the assessment criteria of winding products including multiple performances need to be considered. How to select a satisfactory tape winding parameter range aimed at two different objects including a physical property and a mechanical performance, this is a question worth considering.

In this article, global multi-parameter and local single-parameter sensitivity analysis are applied respectively to investigate the sensitivity of each parameter in composite tape winding processing. In addition, the study will focus on selecting the tape winding parameter range with the responses of tensile strength and void content. This work may be conducive to guide the composite prepreg tape winding molding process and the selection of technological parameters.

## 2. Tape Winding Process

### 2.1. Composite Prepreg Tape Winding Process

**Figure 1:**
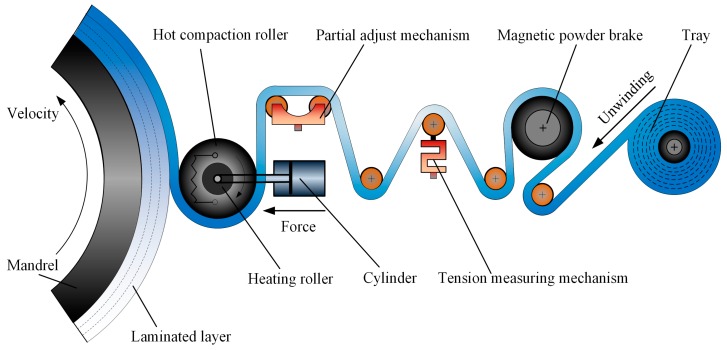
Schematic of the composites prepreg tape winding process.

The composite prepreg tape winding process can be simply described as hoop winding accompanied by superposition of prepreg tape layers. The prepreg tape is a composite of resin matrix and fiber reinforcement. The continuous braided (or unidirectional) fiber bundles are impregnated beforehand by thermoset or thermoplastic resin. In the winding process, tape goes through a hot compaction roller with a certain constant tension, and then combines with the laminated layers on the mandrel [25]. The mandrel rotates with a constant speed and resin is heated to a molten state, simultaneously. As shown in Figure 1, the heat comes from the interior heater strip of the hot compaction roller. Furthermore, the combining area goes by the name of the fusion region. The heating contributes to reducing the velocity of resin and enhances the degree of interlaminar contact. In the fusion region, the hot compaction roller exerts a positive force on the tape. On the one hand, it makes the tape and laminated layers contact intimately. On the other, it contributes to reducing the void content by decreasing the bubble between the interlaminar contact interfaces. A more appropriate winding tension will provide improved strength and fatigue performance for composite tape winding products. Besides, the performance evaluation of winding products, such as compactness and bonding strength, is also subjugated to the mandrel velocity. In brief, a more reasonable parameter interval optimization is of great importance for the composite tape winding process.

### 2.2. Tensile Strength and Void Content Model

Tensile strength and void content are two significant performance indexes for the composite tape winding product. On the one hand, the tensile strength is selected as an evaluation indicator for the mechanical properties of the winding products. On the other hand, the void content is picked as the assessment criteria for the physical performance of the winding products. These two indexes will be combined to evaluate the composite tape winding process. For the uncertainty of the tape winding process, up to now, an ideal and accurate model describing the objective law between the winding process and technological parameters, has not been established. In our work, we needed to build a mathematical simulation between parameters and responses via some well-developed fitting method. When simulations require an amount of time to evaluate a design point, meta models, also called surrogate models or an approximation model, are created to describe the complex system by a simpler and quicker way. Kriging function, neural networks such as RBF (radial basis functions) and the polynomial regression response surface model are examples of approximation methods [26]. As the corresponding argument vectors of the Kriging function need to be calculated by maximum likelihood estimation, so the method seems to become too complicated to calculate parameter estimation. For the neural networks method, a large number of experimental data groups are necessary to train the simulation model. According to Weierstress’ Theorem for Best Polynomial Approximation, almost any type of function can be approximated by polynomial. To reduce the cost of computing, in practice, we always use the second order polynomial regression to simulate and analyze a complex system. However, the second order polynomial regression model is not applicable to fit to the extra high order nonlinear model.

To make the model more generic, in this article, the second order polynomial regression model was chosen to reveal the changing rule of tensile strength and void content. The polynomial regression model on the effect of process parameters on void content or tensile strength are given as:

(1) $$T_{S}(V_{C})=f(x_{1},x_{2},\ldots ,x_{n})=\alpha_{0}+\sum_{i=0}^{n}\alpha_{i}x_{i}+\sum_{i=0,j=0}^{n}\alpha_{ij}x_{i}x_{j}+\sum_{i=0}^{n}\alpha_{ii}x_{i}^{2}$$

where *T_S_* and *V_C_* are tensile strength model and void content model, respectively; *x_i_* is the ith winding process parameter, and *α_i_* are the polynomial coefficients. The study of the process parameters sensitivity analysis method will be based on the polynomial regression model.

## 3. Experiment Procedure and Results

### 3.1. Experiment Design and Sample Preparation

In the composite tape winding process, technological parameters including heating temperature, tape tension, roller force, and winding speed are selected as independent variables; meanwhile, tensile strength and void content are tested separately as the evaluation index. Furthermore, the variable ranges of each technological parameter were set as the actual processing requirements. To save the cost of the experiments, the four-factor and three-level Box–Behnken design (BBD) based on response surface methodology theory was operated to reveal the tensile strength and void content changing with different process parameter combinations. In addition, results and more details of the experiment are provided in Table 1.

Carbon fiber/epoxy resin composite prepreg tape produced by Gloway Composite Materials Co., Ltd. (Weihai, China). was employed in this experiment. The reinforcement of prepreg tape is with carbon fiber T-300 and the matrix is epoxy resin YH-69. The prepreg tape has a weaving structure with a braided angle of 0°/90°, namely orthogonal braid tape. The size of the tape is 80 mm in width and 0.25 mm in average thickness. The fiber volume fraction is approximately 56 ± 2%. The experiments were implemented in an environment at ambient temperature of 20 ± 2 °C and relative humidity of 25 ± 2%.

**Figure 2:**
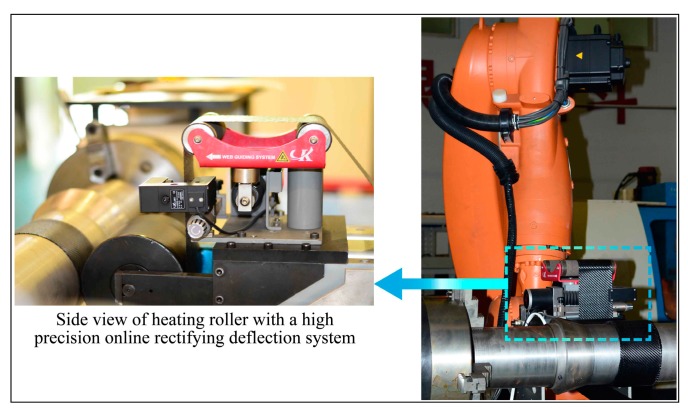
Automate tape winding KUKA robot (XGD-1200).

In the experiment, the tape winding operation was implemented by Automate Tape Winding KUKA Robot (XGD-1200) (KUKA, Augsburg, Germany), as shown in Figure 2. The mandrel used in the winding process is a cylindrical steel column with external diameter of 150 mm. Hoop winding with superposition by layers was utilized to fabricate the specimen, and a high precision online rectifying deflection system was introduced to maintain accurate winding. In the winding process, the hot compaction roller exerted a positive force on the laminates and provided heat for the tape. The hot compaction roller adopted in the experiment is a cylindrical 45 steel column with 160 mm external diameter and 150 mm width.

Ultimately, the winding components produced by hoop winding were cured in an autoclave TEDA Industrial Equipment Co., Ltd. (Tianjin, China). In the curing process, the heat-up rate and curing pressure are 2.5 °C/min and 0.15 MPa, respectively. Meanwhile, the curing temperature should be kept for 150 min once the temperature reaches 150 °C.

### 3.2. Measurement Method

#### 3.2.1. Tensile Strength

**Figure 3:**
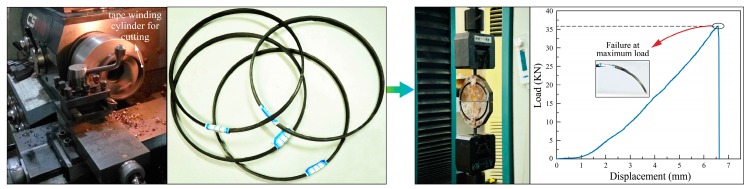
Experiment test samples machining and tensile strength testing process.

Tensile Strength is one of the crucial ways to identify the quality of composite tape winding products. In this paper, the GB/T 1458-2008 standard [27] was employed to develop the tensile strength testing of composite tape winding products. First, each cured ring specimen was mechanically cut along the vertical direction of the fiber axis to obtain the standard testing ring (STR). The dimension of STR is the inner diameter with 150 ± 0.2 mm, width with 6 ± 0.2 mm, and thickness with 3 ± 0.1 mm. Then, the electronic universal testing machine DDL100, manufactured by Changchun Research Institute for Mechanical Science Co., Ltd. (Changchun, China), was utilized to test the tensile strength of composite STR under maximum load. The technological process of STR machining and tensile strength testing can be seen in Figure 3. Finally, the tensile strength of fiber reinforced composite was computed by

(2) $$\sigma_{t}=\frac{F_{b}}{2b\cdot h}$$

where *σ_t_* is the tensile strength *T_S_* and *F_b_* is the maximum load; *b* and *h* are the width and thickness of testing samples, respectively.

#### 3.2.2. Void Content

**Figure 4:**
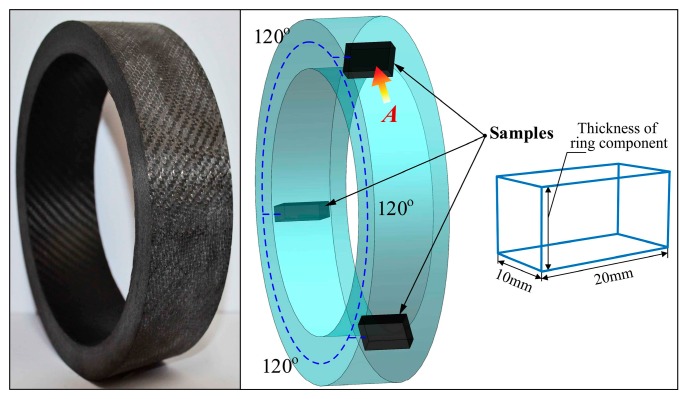
Sketch map of cutting the samples for void content testing.

For composite prepreg tape winding products, the void is a kind of defect which principally exists between the tape layers due to lack of sufficient compaction, air bubble residual, and resin flow. Therefore, the voids have a significant influence on the mechanical performance of composite materials. In the tape winding process, void content can be effectively used to distinguish the physical performance of products. The methods of void content measurement are various such as density measurements, microscopy method [21,28], ultrasonic attenuation [29], and X-ray computed tomography [30], etc. The microscopy method has some advantages like straightforward operation, high precision, and sample calculation. Although it has shortcomings more or less in describing the spatial form of voids in the winding products, the microscopy method is still a good solution for composite material void measurement. As a general type of effective measurement method, micrography was used to evaluate the void content of tape winding products in this paper. Figure 4 shows the photograph of the multi-layered winding component after being cured and turned. According to GB/T 3365-2008 standard [31], three composite samples of a given 20 mm in length, 10 mm in width, and thickness of the ring component in height were cut out from the three isometric positions along the circumference of the ring component (Figure 4). It is worth noting that cracking and stratification were not permitted to appear. A lapping machine was utilized for rough polishing at the first step. Where after, polishing cloth and polishing paste were applied to fine shine the test samples.

**Figure 5:**
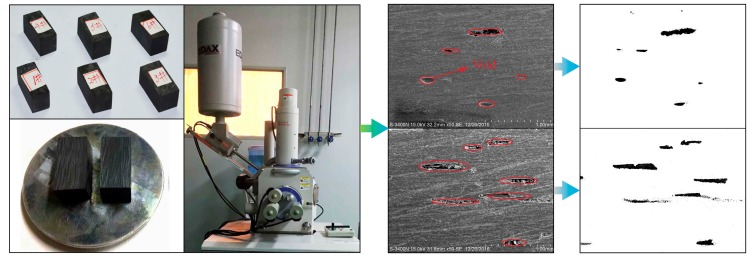
Process of measuring void content for composite tape winding products.

The voids in the prepreg tape winding process principally refer to the holes between the tape layers. In the paper, the unusually lower cavity sizes of voids were ignored for the reason that they were not detectable compared to the relative larger void. The photographs of samples from an orientation of A (Figure 4) were shot by scanning electron microscope (HITACHI S-3400) (Tokyo, Japan) in the experiments. Afterwards, the images were processed by graying and binarizing by using MATLAB (R2017a, The MathWorks Company, Natick, MA, USA). The process of measuring void content for composite tape winding products can be seen in Figure 5. Then the void content was calculated with the ratio of void size to the total sectional area, namely

(3) $$V_{C}=\frac{A_{void}}{A_{total}}\times 100\%$$

where *V_C_* is the void content, %; *A_void_* represents the area of void in the micrograph, mm^2^; and *A_total_* represents the area of total micrograph, mm^2^.

### 3.3. Experiment Results

The tensile strength and void content testing results are combined and shown in Table 1. In this table, *T* is the heating temperature, *F* is the tape tension, *P* is the roller force, and *V* is the winding speed.

The experimental data of tensile strength are analyzed by software of Design-Expert. Then, the polynomial regression model on the effect of process parameters on tensile strength can be obtained as:

(4) $$\begin{array}{cc} T_{S}=f(T,F,P,V)= & -1246.383+22.044T+2.817F+1.534P-2.652V\\ & +6.575\times 10^{-4}T\cdot F-1.316\times 10^{-4}T\cdot P+0.657T\cdot V\\ & -7.635\times 10^{-5}F\cdot P-2.83\times 10^{-3}F\cdot V-2.78\times 10^{-4}P\cdot V\\ & -0.1734T^{2}-4.05\times 10^{-3}F^{2}-4.953\times 10^{-4}P^{2}-3.062V^{2} \end{array}$$

where *T_S_* represents the tensile strength model with a unit of MPa; *T* is the heating temperature, °C; *F* is the tape tension, N; *P* is the roller force, N; and *V* is the winding speed, rpm.

Similarly, the tensile strength representation model can be computed easily as follows:

(5) $$\begin{array}{cc} V_{C}=f(T,F,P,V)= & 10.2-6.564\times 10^{-2}T-6.91\times 10^{-3}F-8.12\times 10^{-3}P+6.203\times 10^{-2}V\\ & -4\times 10^{-5}T\cdot F+2.4\times 10^{-6}T\cdot P-3.74\times 10^{-3}T\cdot V\\ & +1.025\times 10^{-6}F\cdot P+2.75\times 10^{-4}F\cdot V-4\times 10^{-6}P\cdot V\\ & +6.409\times 10^{-4}T^{2}+1.008\times 10^{-5}F^{2}+2.312\times 10^{-6}P^{2}+1.002\times 10^{-2}V^{2} \end{array}$$

where *V_C_* denotes the void content model with a unit of %; *T* is the heating temperature, °C; *F* is the tape tension, N; *P* is the roller force, N; and *V* is the winding speed, rpm.

Furthermore, a series of statistical analysis including analysis of residual, analysis of variance, and predicted versus actual were developed to verify the reliability of the experimental data. Firstly, the normal probability plot of model *T_S_* and *V_C_* showed that both of their points followed an approximate straight line, namely their residuals obeyed a normal distribution. Generally, the experiment parameters have a significant effect on the objective when the *F* value is relative large. According to analysis of variance, the *F* value of model *T_S_* and *V_C_*, 72.48 and 24.68, respectively, both were larger than the benchmark value *F*_0.05_(14, 15) = 2.463. The results showed that the two models were considered to be statistically significant. In addition, the model terms are significant in the case when the *p*-value (the value of ‘Prob > *F*’) of the model is less than 0.05. In this work, the p-value of model *T_S_* and *V_C_* both were less than 0.0001. Furthermore, the ‘R-squared’ can be used to evaluate the degree of fitting. For the *T_S_* model, ‘R-squared’ was 0.9374 and for the *V_C_* model, ‘R-squared’ was 0.9133. Meanwhile, the actual versus predicted plot showed that the maximum errors of model *T_S_* and *V_C_* were 2.21% and 3.57%, respectively. According to the above analysis, it can be seen that the representational model *T_S_* and *V_C_* both had good agreement with their respective experiment results. To sum up, the quadratic regression model is reliable to reveal the response values changing mechanism under different process parameter combinations in the composite prepreg tape winding process.

## 4. Sensitivity Analysis Method

### 4.1. Global Multi-Parameter Sensitivity Analysis

Though good agreement can be obtained between the representational model and experiment results, the model is inconvenient in identifying the relative importance of each process parameter. To recognize the relative significance of the parameters involved in the proposed model, the sensitivities of the model results to input parameters need to be evaluated by assigning a range of variations to each parameter and implementing a generalized sensitivity analysis [32,33].

Global Multi-parametric Sensitivity Analysis (GMSA) is a practical method to reveal the sensitivity of void content and tensile strength on various tape winding parameter changes [34,35,36]. The procedure of GMSA can be briefly described as [37,38]:

1. Select the parameters *x*_1_, *x*_2_, …, *x_n_* to be tested respectively.
2. Set the range of each parameter based on the practical production experience.
3. To every parameter, generate a series of *N* independent random numbers *x_i,j_* (*j* = 1, 2, 3, …, *N*) with a uniform distribution within the defined range.
4. Operating the model using selected parameter sets and calculate the objective function values *y_i_*. The objective function values can be calculated from the modeled values.
5. Determine whether the parameter sets are ‘acceptable’ or ‘unacceptable’ by comparing the objective function values to a given criterion (R). Here, the criterion R is given by:
  (6) $$R=\frac{1}{N}\sum_{j=1}^{N}y_{i}$$
6. For each parameter, calculate the cumulative frequency of ‘acceptable’ or ‘unacceptable’ cases, and illustrate the cumulative frequency curves. Evaluate the separated degree Kolmogorov Smirnov distance (*KS*) of the two cumulative frequency distributions curves. For a certain parameter, the larger the value of *KS*, the more sensitive is the corresponding parameter. The *KS* can be written as:
  (7) $$KS=\underset{\xi }{\sup}\left|S_{a}\left(\xi \right)-S_{u}\left(\xi \right)\right|$$

where *S_a_*(*ξ*) and *S_u_*(*ξ*) are values of ‘acceptable’ and ‘unacceptable’ curves at the same independent variable, respectively; *ξ* is a random number from the corresponding parameter.

**Figure 6:**
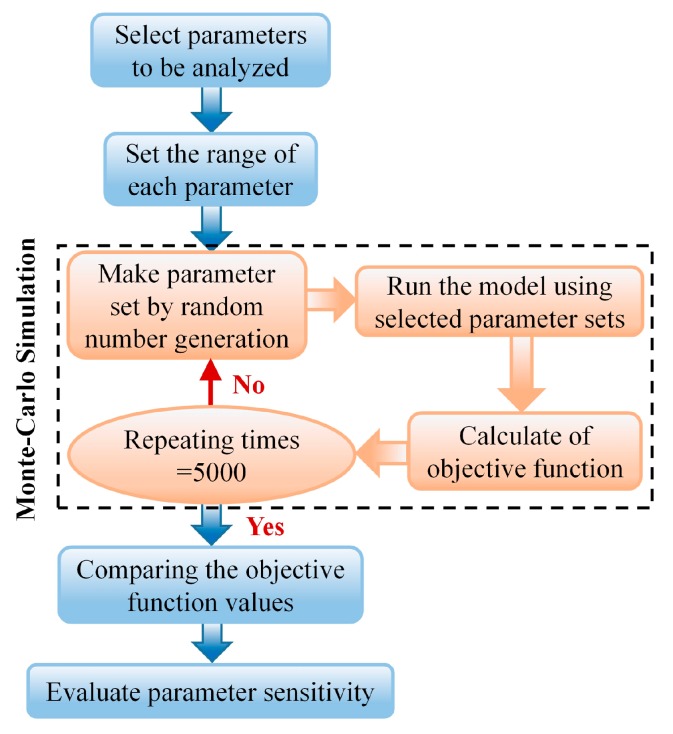
Flow chart of Global Multi-parametric Sensitivity Analysis (GMSA).

Conclusively, the flowchart of GMSA is shown in Figure 6.

### 4.2. Local Single-Parameter Sensitivity Analysis

The relative sensitivity of all process parameters can be comprehensively obtained by the GMSA method. Moreover, a Local Single-parameter Sensitivity Analysis (LSSA) method should be employed to reveal the influence mechanism of each parameter change on evaluation index [39].

Parameter sensitivity refers to the sensitive degree of the evaluation index to each design variable change. The LSSA aims to identify the significant or weak situation where the design variables change affects the objective function. Finally, the ultimate goal of LSSA is to effectively control and optimize the variable parameter and obtain the most ideal objective function [40,41,42]. In the article, the LSSA method is used to investigate the dependence of model outputs with respect to model input variations around a local point in the parameter space, which are quantified by the sensitivity coefficients. Mathematically, the sensitivity coefficients are the first-order derivatives of model outputs with respect to the model parameters [43,44]. Let *S*(*x_i_*) denote the sensitivity coefficients, we can then obtain

(8) $$S(x_{i})=\underset{\Delta x_{i}\to 0}{\lim}\frac{f\left(x_{i}+\Delta x_{i}\right)-f\left(x_{i}\right)}{\Delta x_{i}}=\frac{\partial f(x)}{\partial x_{i}}$$

where *f*(*x*) is the model output and x*_i_* (*i* = 1, 2, 3, …, *n*) is the model input parameter.

As we know, the exponential model of tensile strength was fitted by the BBD experiment which is composed of a series of discrete points. Therefore, when one of the parameter sensitivity coefficients is calculated, the other parameters are substituted by the average value of the respective range. In the following Formula (9), by adding the absolute value function for intuitive, the sensitivity model of the tensile strength on each of the winding parameters (temperature, tension, force, speed) can be presented as:

(9) $$\left\{ \begin{array}{l} S_{S_{T}}^{T}=\left|\frac{\partial f(T,\bar{F},\bar{P},\bar{V})}{\partial T}\right|\\ S_{S_{T}}^{F}=\left|\frac{\partial f(\bar{T},F,\bar{P},\bar{V})}{\partial F}\right|\\ S_{S_{T}}^{P}=\left|\frac{\partial f(\bar{T},\bar{F},P,\bar{V})}{\partial P}\right|\\ S_{S_{T}}^{V}=\left|\frac{\partial f(\bar{T},\bar{F},\bar{P},V)}{\partial V}\right| \end{array}\right.$$

where *T*, *F*, *P*, *V* are heating temperature, tape tension, roller force, and winding speed, respectively; $\bar{T},\;\bar{F},\;\bar{P},\;\bar{V}$, are the mid-point of each process parameter’s range.

Similarly, we can give the sensitivity coefficients of void content on each winding parameter as follows:

(10) $$\left\{ \begin{array}{l} S_{V_{C}}^{T}=\left|\frac{\partial f(T,\bar{F},\bar{P},\bar{V})}{\partial T}\right|\\ S_{V_{C}}^{F}=\left|\frac{\partial f(\bar{T},F,\bar{P},\bar{V})}{\partial F}\right|\\ S_{V_{C}}^{P}=\left|\frac{\partial f(\bar{T},\bar{F},P,\bar{V})}{\partial P}\right|\\ S_{V_{C}}^{V}=\left|\frac{\partial f(\bar{T},\bar{F},\bar{P},V)}{\partial V}\right| \end{array}\right.$$

## 5. Sensitivity Analysis Results

### 5.1. Global Multi-Parameter Sensitivity Analysis Results

The global multi-parameter sensitivity analysis was performed utilizing the Monte-Carlo simulation method exactly as the flow chart shown in Figure 6. The repeating time was set at 5000. The resulting cumulative frequency distributions of acceptable and unacceptable cases, which are divided by comparing subjective criteria and objective function values, are drawn in the manner of two separated curves. The solid lines and dashed lines represent cumulative frequency distributions of the acceptable and unacceptable cases, respectively. If the two distributions are not statistically different, the parameter is classified as insensitive; otherwise, the parameter is classified as sensitive. The values of statistical *KS* are compared to each other and then the sensitive or insensitive parameters are divided.

**Figure 7:**
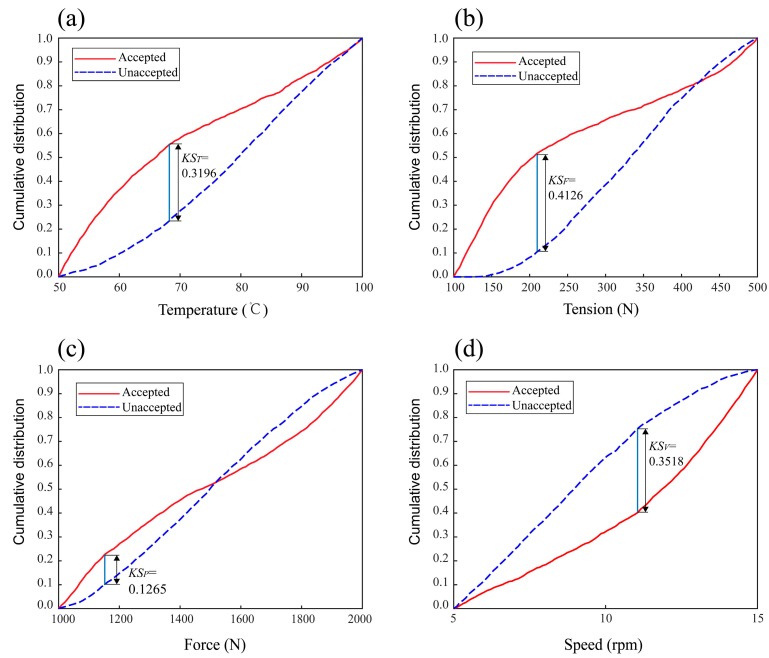
Results of GMSA for the tensile strength model. (**a**) Temperature, (**b**) Tension, (**c**) Force, (**d**) Speed.

**Figure 8:**
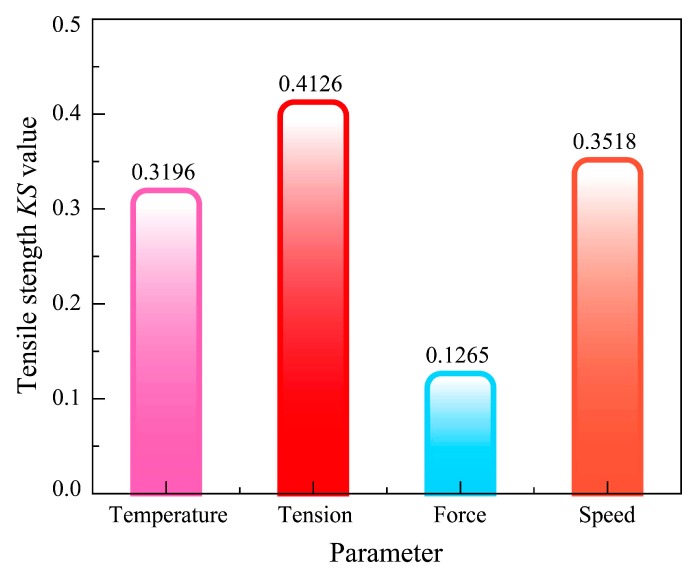
Cartogram of *KS* values for the tensile strength model.

Results of GMSA for parameters of the tensile strength model are shown in Figure 7. From the diagram, we know the separated degree of each parameter including temperature, tension, and force, while the speed distribution curves are *KS_T_* = 0.3196, *KS_F_* = 0.4126, *KS_P_* = 0.1265, *KS_V_* = 0.3518. The statistical data of *KS* values are presented in Figure 8, for intuition and comparison. The results show that the tensile strength of composite tape winding products is most sensitive to the variation of tape tension, next sensitive to the winding speed and heating temperature, and least sensitive to roller force. As the *KS* value of force is small when compared to the other parameters, here we consider the roller force as an insensitive parameter for the tensile strength model.

**Figure 9:**
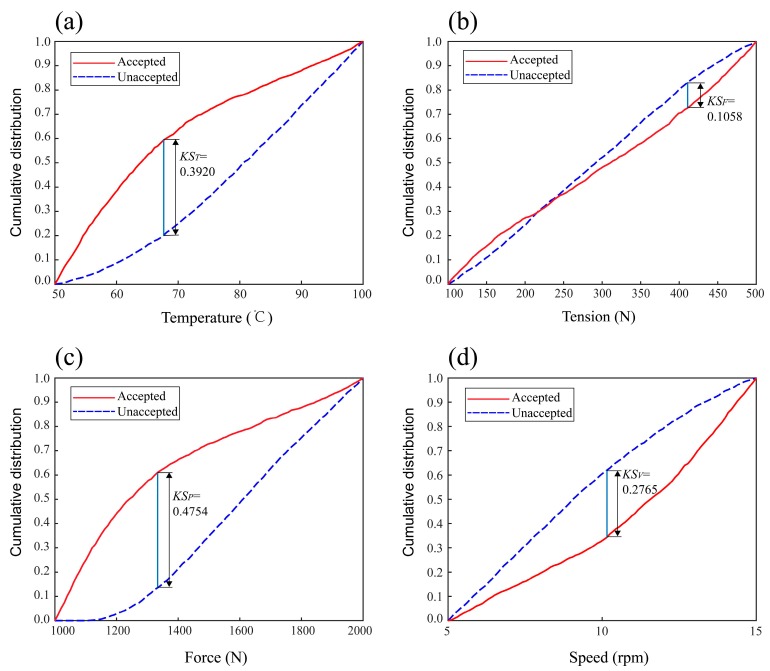
Results of GMSA for the void content model. (**a**) Temperature, (**b**) Tension, (**c**) Force, (**d**) Speed.

**Figure 10:**
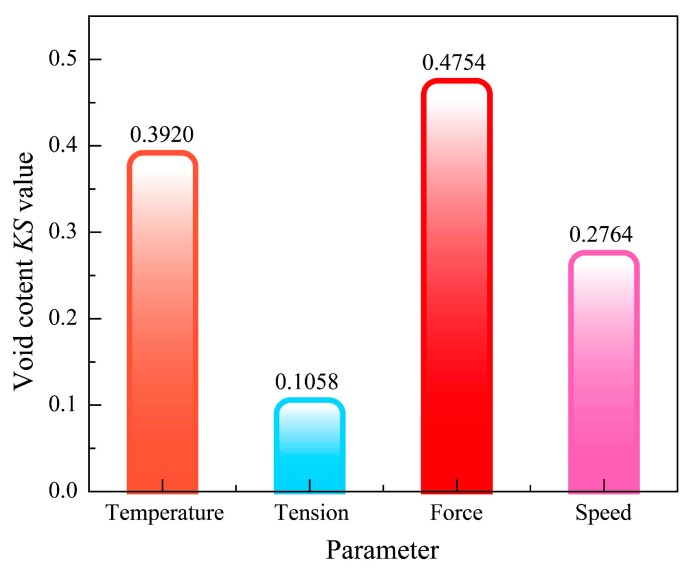
Cartogram of KS values for the void content model.

Figure 9 gives the results of GMSA for parameters of the void content model. From the diagram, the separated degree of each parameter including temperature, tension, force. and speed distribution curves is *KS_T_* = 0.3920, *KS_F_* = 0.1058, *KS_P_* = 0.4754, *KS_V_* = 0.2764. As shown in Figure 10, the *KS* values of the process parameters are obviously different. The results show that the void content of the composite tape winding products is most sensitive to the variation of roller force, next sensitive to heating temperature and winding speed, and least sensitive to tape tension. As the *KS* value of tension is small when compared to the other parameters, finally, we consider the tape tension to be an insensitive parameter for the tensile strength model.

### 5.2. Local Single-Parameter Sensitivity Analysis

According to Formulas (9) and (10), single parameter sensitivities of each process parameters on tensile strength and void content were calculated, respectively. The roller force is an insensitive parameter for the tensile strength model, so the sensitivity of force is not calculated. The same goes for the sensitivity of tape tension in the void content model. The results are shown in Formulas (11) and (12) with a form of absolute value function. The sensitivities of heating temperature, tape tension, roller force, and winding speed are calculated based on the sensitivity model of the single process parameter. The mid-point of each process parameters’ range, namely $\bar{T}$ = 75 °C, $\bar{F}$ = 300 N, $\bar{P}$ = 1500 N, $\bar{V}$ = 10 rpm, are put into the Formulas (9) and (10). Then the sensitivity of each parameter can be written as

(11) $$\left\{ \begin{array}{l} S_{S_{T}}^{T}=\left|-3.468\times 10^{-4}T+28.616\right|\\ S_{S_{T}}^{F}=\left|-8.1\times 10^{-3}F+2.724\right|\\ S_{S_{T}}^{V}=\left|-6.124V+45.372\right| \end{array}\right.$$

(12) $$\left\{ \begin{array}{l} S_{V_{C}}^{T}=\left|1.282\times 10^{-3}T-0.111\right|\\ S_{V_{C}}^{P}=\left|4.625\times 10^{-6}P-7.673\times 10^{-3}\right|\\ S_{V_{C}}^{V}=\left|2.004\times 10^{-2}V-0.142\right| \end{array}\right.$$

**Figure 11:**
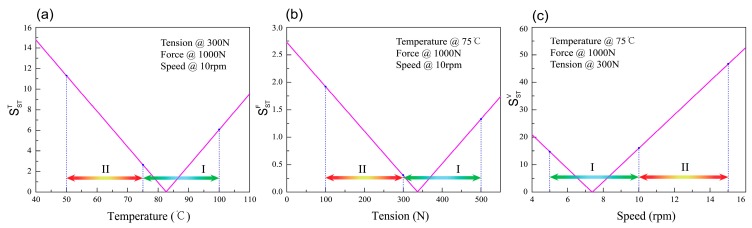
Single-parameter sensitivity curves for process parameters of the tensile strength model. (**a**) Temperature, (**b**) Tension, (**c**) Speed.

**Figure 12:**
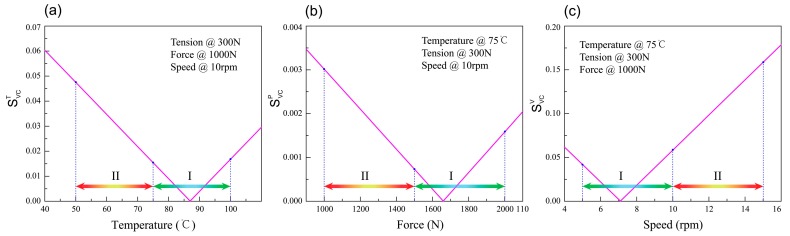
Single-parameter sensitivity curves for process parameters of the void content model. (**a**) Temperature, (**b**) Tension, (**c**) Speed.

Based on the results of local single-parameter sensitivity analysis, Formulas (9) and (10), a series of graphs was drawn as shown in Figure 11 and Figure 12. Figure 11 gives the single parameter sensitivity curve of the tensile strength model. It can be seen from Figure 11c that the sensitivity of winding speed has obviously a large change in scope and the range of the sensitivity curve ordinate value in zone II is larger than zone I. These mean the tensile strength value has a more relative reliable change in zone I. Subsequently, Figure 12 shows the results of the single parameter sensitivity curve of the void content model. The sensitivity curve ordinate values of temperature and speed change greatly between range I and II, as shown in Figure 12a,b.

### 5.3. Stability and Instability Interval Division

The stability interval of process parameters is where the *T_S_* or *V_C_* is insensitive to the change of parameter and the instability interval means that the *T_S_* or *V_C_* is sensitive to the change of parameter in this range. For the insensitive parameter, we consider the whole parameter range as the stability interval but for the sensitive parameter, we must divide the stability and instability interval of the parameter range.

According to the response surface experiments, the experiment was designed according to four factors and three levels testing table. For each sensitive parameter, first, divide the parameter range into two intervals, (*M*_1_, *M*_2_), (*M*_2_, *M*_3_). According to sensitivity curves, calculate the sensitivity mean values in the range of (*M*_1_, *M*_2_) and (*M*_2_, *M*_3_), which are denoted by *A*_1_, *A*_2_. Afterwards, calculate the mean value of *A*_1_, *A*_2_ (denoted by *A*_0_). If *A_i_* < *A*_0_ (*i* = 1, 2), the range of (*M_i_*, *M_i_*_+1_) is considered as a stability interval; otherwise, the range is instability.

The stability and instability intervals of each process parameter are drawn in Figure 11 and Figure 12. In the pictures, two kinds of ranges are distinguished utilizing different colored arrows. Section I represents the stability interval and Section II represents the instability interval. Finally, the stability and instability intervals of the tensile strength and void content model’s parameter ranges are shown in Table 2 and Table 3, respectively.

## 6. Interval Optimization

**Figure 13:**
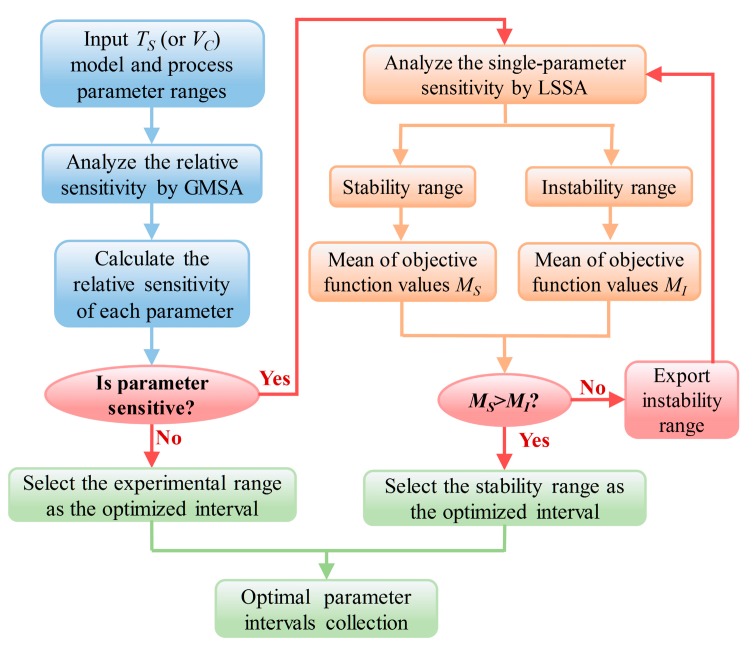
Flow chart of parameter interval optimization process.

The stability and instability range of each process parameter we obtained are perhaps not the best results. Therefore, it is necessary to further optimize the parameter intervals. The method of optimization is demonstrated in Figure 13.

For insensitive parameters, select the experimental range as the optimized interval but for sensitive parameters, analyze the single-parameter sensitivity and then divide the stability and instability intervals. More remarkable, these steps are imperative for a sensitive parameter’s interval optimization. First of all, generate 5000 random parameter sets in GMSA and the corresponding objective function values (*T_S_* or *V_C_*), compute the mean value of those objective function values in the stability (*M_S_*) and instability interval (*M_I_*). If the value of *M_S_* is greater than *M_I_*, the stability range is identified as an optimized interval; otherwise, the instability range is classified as an optimized interval. For the case the optimized interval is the instability range, divide the instability range into two regions. Meanwhile, determine a smaller region of stability and instability range, and return to the first step to continue the calculation until the stability range is found as optimized interval.

### 6.1. Tensile Strength Model

As can be seen, Table 4 shows the interval optimization procedure for process parameters of the tensile strength model. In the first round of optimization, the mean objective values (i.e., tensile strength) of three parameters, including temperature, tension, and speed, were calculated and compared, respectively. Nevertheless, the larger mean objective value of temperature and tension lay within the instability range. As a result, the instability ranges of temperature and tension were classified as the optimized interval, and the stability range of speed was classified as the optimized interval. According to the flow chart of the parameter interval optimization process (Figure 13), further division and optimization would be required for the temperature and tension. In the second round of optimization, the mean objective value of the stability range is greater than the instability range for the temperature and tension, hence the stability ranges of temperature and tension were classified as the optimized interval. Table 5 displays the final results of interval optimization for process parameters of the tensile strength model.

### 6.2. Void Content Model

For the void content model, the lower the value of mean objective values, the better the performance of the winding products. Table 6 shows the interval optimization procedure for process parameters of the void content model. In the first round of optimization, the mean objective values (i.e., void content) of three parameters, including temperature, force, and speed, were calculated and compared, respectively. However, the lower mean objective value of temperature lay within the instability range. As a result, the instability range of temperature (50 °C, 75 °C) was categorized as the optimized interval, and the stability ranges of force and speed were classified as the optimized interval. According to Figure 13, further division and optimization would be required for the temperature. In the second round of optimization, the mean objective value of the stability range (62.5 °C, 75 °C) is lower than the instability range (50 °C, 62.5 °C) for the temperature, so the stability range of temperature (62.5 °C, 75 °C) was classified as the optimized interval. Table 7 provides us with the final results of interval optimization for the process parameters of the tensile strength model.

### 6.3. Comprehensive Optimized Interval of Winding Parameters

According to the interval optimization for the process parameters of tensile strength and void content model, we obtained the comprehensive optimized interval of the composite tape winding process parameters as follows: heating temperature within (62.5 °C, 75 °C), tape tension within (200 N, 300 N), roller force within (1500 N, 2000 N) and winding speed within (5 rpm, 10 rpm). The specific details of the optimization results are identified in Table 8.

### 6.4. Experimental Verification

In the verification test, six groups of the process parameter sets were selected randomly from the optimized intervals. In Table 9, the former three groups (i.e., the 1st, 2nd, 3rd groups) and the later three groups (i.e., the 4th, 5th, 6th groups) of the process parameter sets were selected randomly from inside and outside of the optimized intervals, respectively. The simulation and experimental objective value (tensile strength and void content) of each parameter sets were calculated and recorded one by one. According to Table 9, both the tensile strength and void content of the winding specimens, which were produced with the process parameter sets in the optimized intervals, are better than those out of the optimized range. Meanwhile, the testing results indicate that the error between simulation value and experiment data did not exceed 5%. Consequently, the correctness and validation of the simulation model were demonstrated once again.

## 7. Summary

The paper presented an integrated methodology by combining the multi-parameter relative sensitivity analysis and the single-parameter sensitivity analysis. Global multi-parameter and local single-parameter sensitivity analysis were applied respectively to investigate the sensitivity of each parameter in the composite tape winding processing. Finally, the authors provided the optimized comprehensive intervals of the tape winding parameters. Meanwhile, the verification test validated that the optimized intervals of the process parameters were reliable and stable for winding products manufacturing.

The results of GMSA show that the tensile strength of composite tape winding products is most sensitive to the variation of tape tension, next sensitive to winding speed and heating temperature, and least sensitive to roller force. As a result, we considered the roller force to be an insensitive parameter for the tensile strength model. Meanwhile, the void content of the composite tape winding products is most sensitive to the variation of roller force, next sensitive to heating temperature and winding speed, and least sensitive to tape tension. Finally, we considered the tape tension to be an insensitive parameter for the tensile strength model.

Thereafter, single parameter sensitivities of each process parameter on tensile strength and void content were calculated, respectively. The sensitivities of heating temperature, tape tension, roller force, and winding speed were calculated based on the sensitivity model of the single process parameter.

According to the optimization process, the optimized intervals of the process parameters are: heating temperature within (62.5 °C, 75 °C), tape tension within (200 N, 300 N), roller force within (1500 N, 2000 N), and winding speed within (5 rpm, 10 rpm). The proposed and validated parameter intervals presented in this work may well contribute to increasing the knowledge of the composite tape winding process.

## Figures and Tables

**Table 1:** Experiment and measurement results.

No.	Process Parameters				Objective Value	
	Temperature *T* (°C)	Tension *F* (N)	Force *P* (N)	Speed *V* (rpm)	Tensile Strength (MPa)	Void Content (%)
1	75	300	1000	15	896.92	1.64
2	50	300	2000	10	942.36	1.19
3	100	300	2000	10	1012.24	0.25
4	75	500	1500	15	951.33	1.26
5	75	300	1500	10	1215.31	0.13
6	75	300	2000	5	1123.47	0.35
7	100	500	1500	10	1062.27	0.73
8	50	500	1500	10	920.61	1.45
9	75	300	1500	10	1212.53	0.15
10	75	300	1500	10	1209.32	0.17
11	75	500	1000	10	1029.13	1.39
12	75	100	1500	5	967.14	0.95
13	75	500	2000	10	1018.74	0.71
14	100	100	1500	10	951.72	0.87
15	75	100	2000	10	866.58	0.59
16	75	300	1500	10	1213.24	0.14
17	75	300	1500	10	1210.68	0.15
18	100	300	1500	15	1130.29	0.28
19	50	100	1500	10	823.21	0.79
20	75	100	1500	15	876.68	0.38
21	75	100	1000	10	846.43	1.68
22	50	300	1500	5	1116.38	0.31
23	50	300	1500	15	783.26	2.19
24	75	300	2000	15	911.07	1.16
25	100	300	1500	5	1134.81	0.27
26	100	300	1000	10	997.85	1.06
27	75	500	1500	5	1053.11	0.73
28	50	300	1000	10	921.39	2.12
29	75	300	1000	5	1106.54	0.79

**Table 2:** Stability and instability interval for process parameters of the tensile strength model.

Process Parameter	Stability Interval	Instability Interval
Temperature (°C)	(75 °C, 100 °C)	(50 °C, 75 °C)
Tension (N)	(300 N, 500 N)	(100 N, 300 N)
Force (N)	(1000 N, 2000 N)	
Speed (rpm)	(5 rpm, 10 rpm)	(10 rpm, 15 rpm)

**Table 3:** Stability and instability interval for process parameters of the void content model.

Process Parameter	Stability Interval	Instability Interval
Temperature (°C)	(75 °C, 100 °C)	(50 °C, 75 °C)
Tension (N)	(100 N, 500 N)	
Force (N)	(1500 N, 2000 N)	(1000 N, 1500 N)
Speed (rpm)	(5 rpm, 10 rpm)	(10 rpm, 15 rpm)

**Table 4:** Interval optimization for process parameters of the tensile strength model.

Parameters	Range Division	Mean Objective Value (%)	Stability
**The First Round of Optimization (5000 times)**			
Temperature	(50 °C, 75 °C)	1035.333	instability range
	(75 °C, 100 °C)	961.699	
Tension	(100 N, 300 N)	1054.731	instability range
	(300 N, 500 N)	944.352	
Speed	(5 rpm, 10 rpm)	1014.125	stability range
	(10 rpm, 15 rpm)	983.493	
**The Second Round of Optimization (5000 times)**			
Tension	(100 N, 200 N)	1045.158	
	(200 N, 300 N)	1057.711	stability range
Temperature	(50 °C, 62.5 °C)	1023.512	
	(62.5 °C, 75 °C)	1049.634	stability range

**Table 5:** Results of interval optimization for process parameters of the tensile strength model.

Process Parameter	Optimized Interval
Temperature (°C)	(62.5 °C, 75 °C)
Tension (N)	(200N, 300N)
Force (N)	(1000 N, 2000 N)
Speed (rpm)	(5 rpm, 10 rpm)

**Table 6:** Optimization of the tape winding process parameters of the void content model.

Parameters	Range Division	Mean Objective Value (%)	Stability
**The First Round of Optimization (5000 times)**			
Temperature	(50 °C, 75 °C)	0.5207	instability range
	(75 °C, 100 °C)	0.8418	
Force	(1000 N, 1500 N)	0.8782	
	(1500 N, 2000 N)	0.5518	stability range
Speed	(5 rpm, 10 rpm)	0.5235	stability range
	(10 rpm, 15 rpm)	0.8951	
**The Second Round of Optimization (5000 times)**			
Temperature	(50 °C, 62.5 °C)	0.6918	
	(62.5 °C, 75 °C)	0.4807	stability range

**Table 7:** Process parameters interval optimization.

Process Parameter	Optimized Interval
Temperature (°C)	(62.5 °C, 75 °C)
Tension (N)	(100 N, 500 N)
Force (N)	(1500 N, 2000 N)
Speed (rpm)	(5 rpm, 10 rpm)

**Table 8:** Comprehensive optimized interval of winding parameters.

Process Parameter	Optimized Interval
Temperature (°C)	(62.5 °C, 75 °C)
Tension (N)	(200 N, 300 N)
Force (N)	(1500 N, 2000 N)
Speed (rpm)	(5 rpm, 10 rpm)

**Table 9:** Experimental verification of optimized intervals.

No.	*T* (°C)	*F* (N)	*P* (N)	*V* (rpm)	Tensile Strength (MPa)		Void Content (%)	
					Simulation	Experiment	Simulation	Experiment
1	65	252	1621	6.8	1190.81	1220.57	0.139	0.14
2	69	235	1580	9.1	1167.96	1138.42	0.168	0.18
3	72	284	1874	7.2	1159.43	1195.61	0.148	0.16
4	58	373	1275	12.5	998.69	1002.84	1.427	1.38
5	79	169	1480	13.7	1012.48	996.70	0.523	0.57
6	92	452	1341	14.5	1032.12	1021.39	0.876	0.95
